# Chiral Recognition of D/L-Ribose by Visual and SERS Assessments

**DOI:** 10.3390/molecules28186480

**Published:** 2023-09-07

**Authors:** Guohua Yao, Chao Liu, Shereen M. Elsherbiny, Qing Huang

**Affiliations:** 1 The Education Ministry Key Laboratory of Resource Chemistry, Joint International Research Laboratory of Resource Chemistry of Ministry of Education, Shanghai Non-carbon Energy Conversion and Utilization Institute, Shanghai Normal University, Shanghai 200234, China; ghyao@shnu.edu.cn; 2 CAS Key Laboratory of Ion-Beam Bioengineering, Institute of Intelligent Machines, Hefei Institutes of Physical Science, Chinese Academy of Sciences, Hefei 230031, China; aliza97@mail.ustc.edu.cn (C.L.);

**Keywords:** ribose, chiral recognition, visual sensor, colorimetry, SERS

## Abstract

Ribose is the central molecular unit in ribose nucleic acid (RNA). Ribose is a key molecule in the study of many persistent scientific mysteries, such as the origin of life and the chiral homogeneity of biological molecules. Therefore, the chiral recognition of ribose is of great significance. The traditional method of chiral recognition of ribose is HPLC, which is time-consuming, expensive, and can only be operated in the laboratory. There is no report on optical analytical techniques that can quickly detect the chirality of ribose. In this study, a simple and convenient approach for the chiral recognition of ribose has been developed. β-cyclodextrin(β-CD)-coated Ag NPs aggregate after adding D-ribose, so that D-/L-ribose can be identified using visual colorimetry and/or surface-enhanced Raman spectroscopy (SERS). The color change visible to the naked eye can readily distinguish the chirality of ribose, while the SERS method can provide the more sensitive analysis of enantiomeric ribose. The advantages of this method are that it is fast, convenient, low cost, and can be operated outside the laboratory. DFT calculations show that D-ribose and cyclodextrin have the same chirality, forming multiple strong hydrogen bonds between them; thus, D/L-ribose will induce different optical effects.

## 1. Introduction

Ribose, a chiral five-carbon sugar molecule, is well known as the central molecular unit in ribose nucleic acid (RNA) and the precursor of deoxyribose to construct DNA [1]. Ribose is related to the origin of life, since ribose and its sister pentoses (arabinose, xylose, and lyxose) can be made under alkaline conditions from simple organic precursors (formaldehyde and glycolaldehyde) known to exist in interstellar space and presumably available on early Earth [1,2,3]. As the general notion of an “RNA World hypothesis” [4], in the early development of life on the Earth, genetic continuity was assured by the replication of RNA, and ribose is believed to be the first genetic polymer for the emergence of life [5,6]. The origin of the natural selection of ribose as the exclusive sugar component for RNA is also one of the greatest mysteries of the origins of life [6].

**Figure 1:**
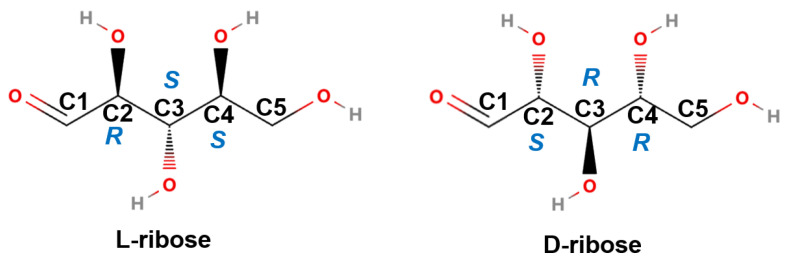
Structures of L-ribose and D-ribose. The chiralities of C2, C3, and C4 for L-ribose are *R*, *S*, and *S*, respectively, while D-ribose has opposite chirality.

In the entire biological system on Earth, almost all biological molecules are chiral, such as L-amino acids and D-sugars, and the chiral macromolecules they make up. Ribose formed under interstellar–analog conditions has both D- and L-enantiomers, while only D-ribose is found in RNA in organisms [1]. The structures of L-ribose and D-ribose are shown in Figure 1. The origin of life, based on the homochirality of biomolecules, is a persistent mystery [1,7]. Did life begin by using both forms of chirality and then one of the forms disappeared [7]? In the “RNA world hypothesis”, RNA is the genetic material, while ribose is the only chiral fragment molecule in RNA. Once an inherent preference for one chiral form over another has been established, chirality is propagated to downstream molecular products (including lipids and other important metabolites) resulting from complex biosynthetic pathways from the chemical principles of chiral induction and diastereomeric selection [8,9]. Therefore, the chiral homogeneity of biological molecules may be related to the chirality of ribose. Many studies have tried to understand the chirality of ribose. For instance, two enantiomeric forms related to the ribose ring have been studied in an autocatalytic reaction [8]; the properties of nuclear forces are found to uniquely determine the right sugars (ribose) that strongly dominate (chiral purity) on the Earth [10]; ribose is found to have the viability for mediating enantioenrichment of over 80% of the enantiomeric excess in amino acid precursors [11]. It is obvious that if the chirality of ribose molecules can be traced rapidly or even in situ in these studies, it will greatly promote relevant research.

Therefore, to solve these persistent scientific mysteries, there has been special interest in developing advanced methods for rapid recognition through qualitative and quantitative analyses of the chirality of ribose. On the other hand, enantiomers (like D-ribose and L-ribose) exhibit different or entirely opposite potency, toxicity, transport mechanisms, and pathways of metabolism in organisms [12,13]. Hence, chiral recognition is of great importance, especially in areas of bioanalysis, pharmacy, agricultural chemicals, and analytical chemistry [14,15,16].

Traditionally, the separation and recognition of enantiomers were operated by chromatographic methods [12]. D-ribose and L-ribose can also be separated using the high-performance liquid chromatography (HPLC) method, with the retention time of ribose being nearly 2 h [17]. The disadvantages of HPLC are that it is costly, time-consuming, and inconvenient for examination in the laboratory. Recently, optical analytical techniques, such as colorimetry/UV-Vis, circular dichroism, and fluorescence spectroscopy, have been used to study chiral recognition [18]. The optical methods have many advantages, such as fast analysis, ease of handling, low cost, high sensitivity, and adaptation to automation. And they have been applied to chiral recognition of amino acids and other molecules, such as the colorimetric recognition of alanine [16], pyroglutamic acid [19], glucose [20], and cysteine [15]. Unfortunately, to the best of our knowledge, there are still difficulties in the application of these optical methods to the chiral recognition of ribose, and there is no report on their effective application.

β-CD is composed of seven D-glucose molecules, and the outer surface of a β-CD appears like a truncated cone [21]. Cyclodextrins have different interactions with chiral enantiomers, thus making cyclodextrins important in applications of chiral separation science, such as capillary electrophoresis, gas chromatography, and liquid chromatography. We speculate that cyclodextrin will also have different interactions with DL ribose, thus having different optical effects. Therefore, in this study, we attempted to load cyclodextrin onto silver nanoparticles which exhibit optical properties, such as localized surface plasmonic resonance (LSPR) and a surface-enhanced Raman scattering effect, and to develop an optical method based on surface-enhanced Raman spectroscopy (SERS) combined with a visual guide to the achieve rapid, low-cost, non-laboratory chiral recognition of ribose. In addition, density functional theory (DFT) will be performed to understand the relevant mechanisms.

## 2. Results and Discussion

### 2.1. Chiral Recognition by Visual Assessment

**Figure 2:**
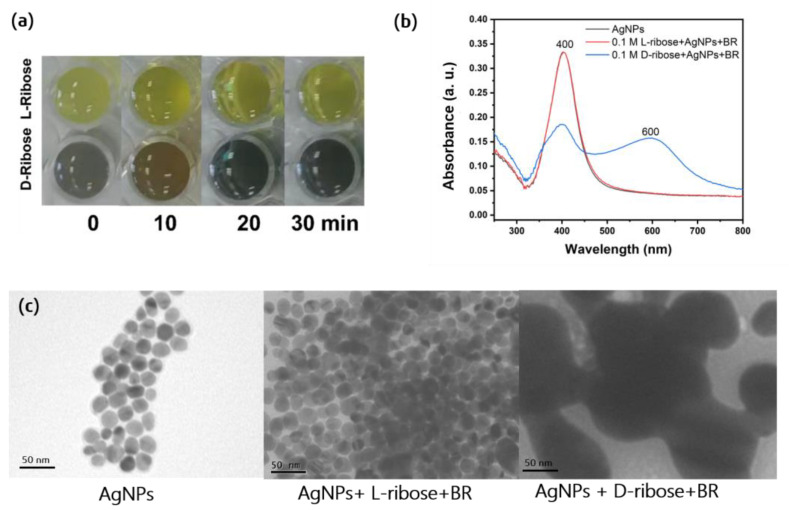
(**a**) The photographs of the mixture of 200 μL Ag@CD NPs (abbreviated as AgNPs in the figures), 200 μL 0.1M L-ribose or D-ribose, 0, 10, 20, 30 min after adding 20 μL BR of pH = 3.2. (**b**) UV–vis absorption spectra of Ag@CD NPs (red curve), a mixture of 0.1 M L-ribose with Ag@CD NPs and BR after 30 min (blue curve), and a mixture of 0.1 M D-ribose with Ag@CD NPs and BR after 30 min (black curve). (**c**) The TEM images of (**left**): Ag@CD NPs, (**middle**): 0.1 M L-ribose with Ag@CD NPs and BR after 30 min, (**right**): 0.1 M D-ribose with Ag@CD NPs and BR after 30 min.

In this strategy of ribose chiral recognition, silver nanoparticles (AgNPs) covered with β-cyclodextrin (β-CD) were first prepared. As shown in Figure 2a, when 0.1 M L-ribose and D-ribose were mixed with silver sol, respectively, the color of the mixed solution did not change and remained the same light yellow as the silver sol. However, after adding Britton–Robinson (BR) with a pH value of 3.2, the color of the D-ribose mixture turned purple–red immediately, and after 30 min, it turned gray–green while the color of the L-ribose mixture remained unchanged. Therefore, we can intuitively distinguish the chirality of ribose by the color of the mixture. However, when only adding BR to beta-CD@Ag Nanoparticles, both the color and UV–vis spectra did not change, as shown in Figure S1. From the UV–vis spectra in Figure 2b, the absorption band of the silver sol and the L-ribose mixture is about 400 nm. They almost have the same absorption curve, indicating that silver nanoparticles in the mixed solution did not aggregate. The TEM images of the nanoparticles are from the same synthesis batch shown in Figure 2c, proving that the D-ribose mixture had aggregated. Therefore, the aggregation of Ag@CD NPs induced by D-ribose changes the optical properties, such as localized surface plasmonic resonance [18], resulted in a decrease in the surface plasmon absorption at 400 nm and the formation of a broadened surface plasmon band at around 600 nm. The β-CD molecule is composed of seven D-glucose molecules; the binding energy between D-ribose and β-CD may be stronger than L-ribose, which would enhance the interaction of β-CDs between different particles. Since the Ag or Au nanoparticles aggregated or their aggregates disassembled, both their color and absorbance values in the UV–vis region changed [18,22].

**Figure 3:**
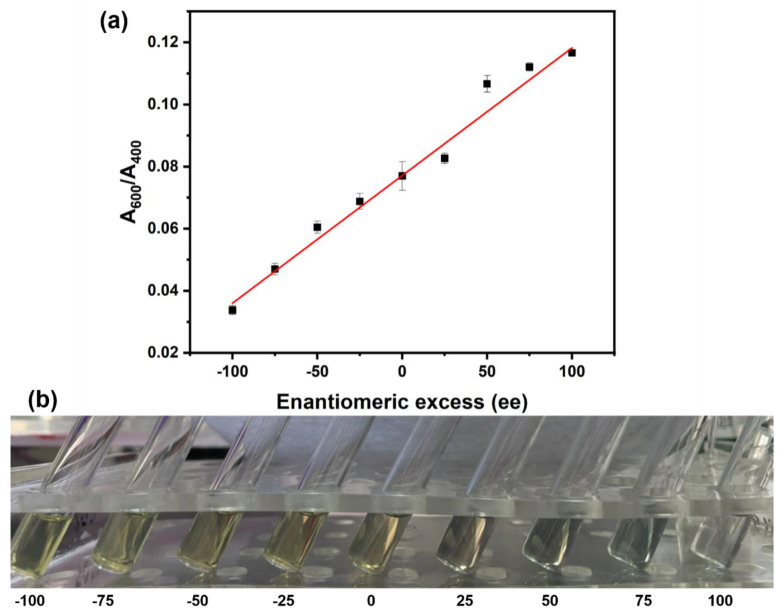
(**a**) The absorbance ratio A600/A400, the data of black dots comes from the average absorbance of 5 repeated measurements, and the error bar comes from the standard deviation generated by 5 repeated measurements; (**b**) The photos, of Ag@CD NPs responding to a series of different enantiomeric excesses (ee) of ribose (−100, −75, −50, −25, 0, 25, 50, 75, 100) after adding BR for 30 min. The sum of concentrations of L-ribose and D-ribose is 0.01 M.

Considering that the absorbance at 600 and 400 nm (A600, A400) are related to the aggregated Ag@CD NPs and the chirality of ribose, the ratio A600/A400 was chosen as the indicator of the chiral recognition of ribose. Figure 3 illustrates the significant difference in the absorbance ratio A600/A400 of Ag@CD NPs responding to a series of different enantiomeric excesses (ee) of ribose (−100, −75, −50, −25, 0, 25, 50, 75, 100), which also correspond to enantiomeric compositions of ribose (L/D = 100:0, 87.5:12.5, 75:25, 62.5:37.5, 50:50, 37.5:62.5, 25:75, 12.5:87.5, 0:100). The sum of the concentrations of L-ribose and D-ribose was 0.01 M. The absorption ratio (A600/A400) increased linearly with the increase in D-ribose composition. The fitted regression equation is y = 0.00041x + 0.077, with a coefficient of determination (R^2^) of 0.990. Since the absorption ratio and content have a good correlation, the adsorption ratio can be used to quantify the ratio of D-ribose and R-ribose, which is particularly important for the research on the origin of life chirality. At the same time, it can also be seen from Figure 3 that with the increase in the D-ribose ratio, the color of the mixture gradually changed from yellow to gray–green. The color of the mixture can also be used to determine the proportion of enantiomers roughly and visually. Two other kinds of typical monose (glucose, mannose) were also detected under the same conditions, but the chirality of these monosaccharides cannot be recognized, as shown in Figure S2. Therefore, this detection strategy is sensitive to ribose.

**Figure 4:**
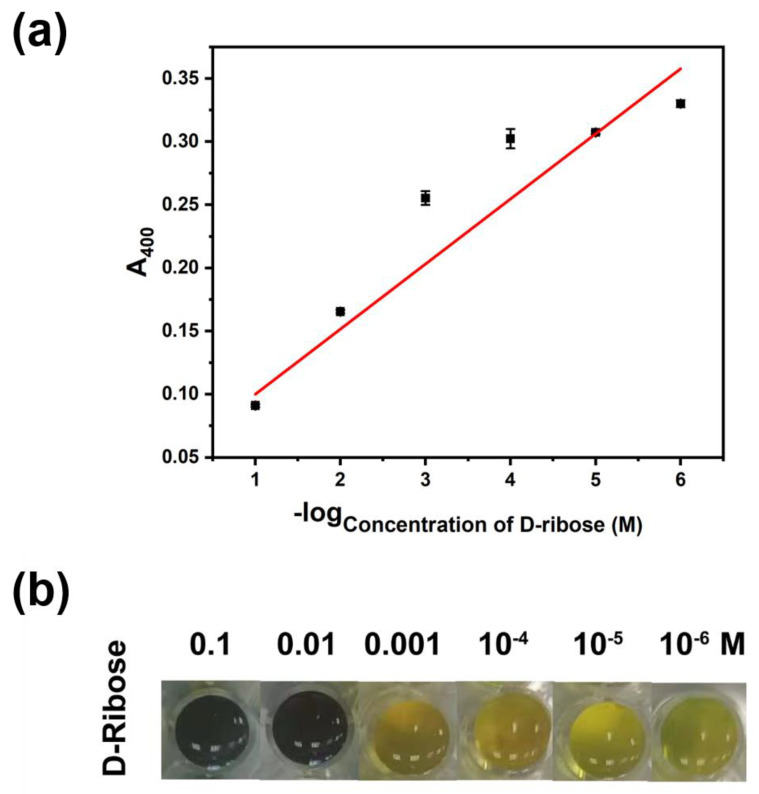
(**a**) The absorbance at 400 nm (A400), the data of black dots comes from the average absorbance of 5 repeated measurements, and the error bar comes from the standard deviation generated by 5 repeated measurements, and (**b**) the photos of Ag@CD NPs responding to a series of different concentrations of D-ribose (from left to right: 0.1, 0.01, 0.001, 10^−4^, 10^−5^, 10^−6^ M) after adding BR for 30 min.

On the other hand, different concentrations of D-ribose were added in this method to test whether Ag@CD NPs can be used for the detection of ribose concentration. As shown in Figure 4, the color of the 0.1 and 0.01 M D-ribose mixture is gray–green and for the 0.001 M D-ribose it is brown. However, the color for the mixtures of 10^−4^ and 10^−5^ M D-ribose is nearly yellow, like the one without D-ribose. Therefore, the limit of detection (LOD) of this visualization discrimination is 10^−3^ M. However, when the concentration of D-ribose increased from 10^−6^ M to 0.1 M, the absorbance of 400 nm regularly decreased according to logarithmic relationship. The fitted regression equation is y = 0.0513x + 0.0480, with a coefficient of determination (R^2^) of 0.975. The A400 value of the mixture of 10^−6^ M D-ribose and Ag@CD NPs is nearly the same as that with blank Ag@CD NPs. With the help of image software, we can obtain the R, G, and B values of the color of samples; the R values to the concentration of D-ribose have a good relationship, as shown in Figure S3. The fitted regression equation is y = 30.49x − 6.28, with a coefficient of determination (R^2^) of 0.990. Directly from the photo information of the sample [23,24], we can estimate the concentration of ribose without the need to measure the spectra of the samples.

### 2.2. SERS Chiral Recognition

**Figure 5:**
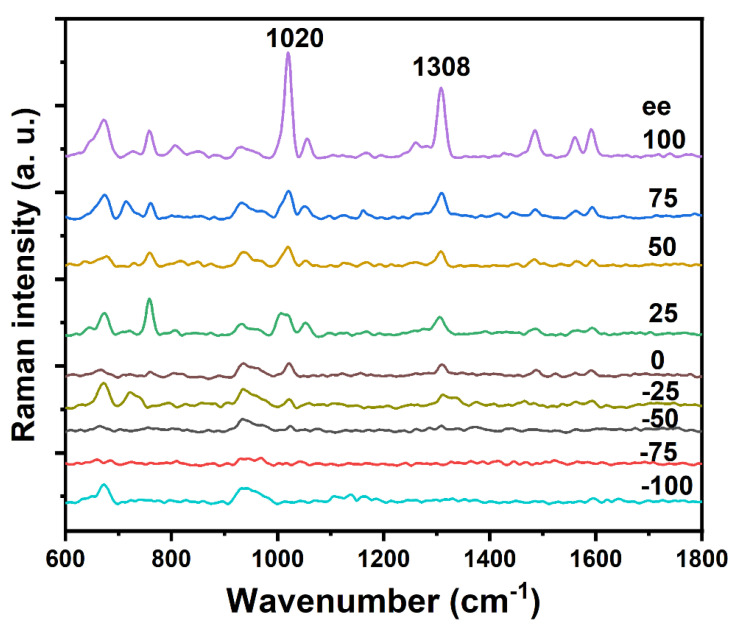
SERS spectra of Ag@CD NPs responding to a series of different enantiomeric excesses (ee) of 0.01 M ribose (−100, −75, −50, −25, 0, 25, 50, 75, 100) after adding BR for 30 min.

The three common optical methods (circular dichroism, absorption/colorimetry, and fluorescence) using Ag/Au nanomaterials have been shown to be sensitive and selective for the detection of various analytes, such as metal ions, small organic compounds, peptides, proteins, and DNA [18]. When compared to circular dichroism and fluorescence sensing system, the UV–vis absorption/colorimetry systems are cheaper and simpler [18]. In this study, we also explore other simple and cheap optical methods for chiral recognition. Silver nanoparticles generally have a surface-enhanced Raman effect, so they could also be used for the chiral recognition of ribose. Figure 5 shows the SERS spectra of Ag@CD NPs responding to a series of different enantiomeric excesses (ee) of ribose (−100, −75, −50, −25, 0, 25, 50, 75, 100) after adding BR for 30 min. The sum of the concentrations of L-ribose and D-ribose was 0.01 M. When the proportion of D-ribose increased, the intensity of the 1020 and 1308 cm^−1^ bands from D-ribose, accordingly, increased. The 1020 cm^−1^ band is mainly attributed to the stretching vibration of the C-O and C-C bonds and the 1308 cm^−1^ band mainly to the bending vibration of the C-H and O-H bonds [25]. The changes in SERS intensity from the analyte are appreciated because of the aggregation. Therefore, these two bands can be used to identify the chirality of ribose. The intensities of the SERS bands at about 680 and 930 cm^−1^ did not show significant changes after the addition of ribose. It can be recognized from Figure S4 that these two bands mainly come from Ag@CD NPs rather than the ribose molecules.

The chiral detection of ribose using surface-enhanced Raman optical activity (SEROA) has been reported [26], which has made a very important contribution for chiral molecular recognition, but there are many differences from our study. First, in the SEROA strategy, the intensity difference is from the Raman scattering of left and right circularly polarized light; therefore, a chiral Raman spectrometer is required. The handheld mobile ROA detection device has not been reported yet, and the ROA measurement usually takes a long time, from a few minutes to tens of hours. Second, there is no difference in the surface-enhanced Raman spectroscopy (SERS) between the two ribose enantiomers: only SEROA has the difference [26]. But in our study, the ordinary SERS of the L/D-ribose already has differences. Third, the difference in the SEROA signal is from the an achiral benzotriazole dye molecule on the Ag NPs, not from the chiral ribose molecules [26]. In our work, the difference in the SERS signal is from chiral ribose molecules, rather than from the β-CD molecule on the Ag NPs.

### 2.3. DFT Calculation and Analysis

**Figure 6:**
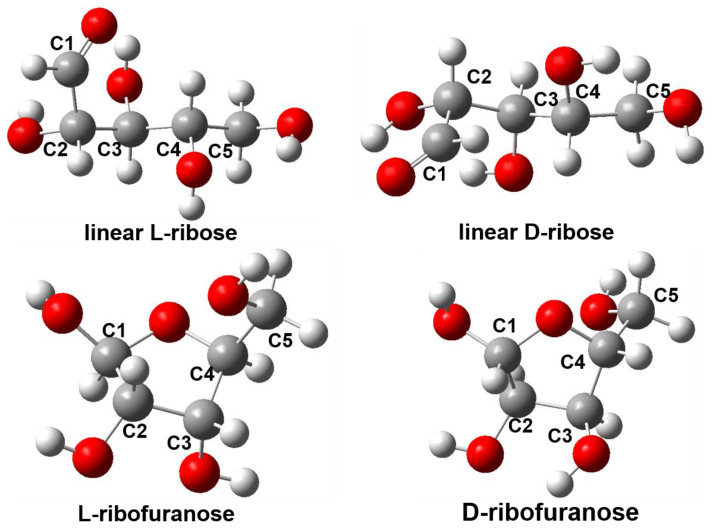
The optimized geometric structures of linear L-ribose, linear D-ribose, L-ribofuranose, and D-ribofuranose, simulated under M06-2X/6-31G(d) level of theory. (Grey: C atom, red: O atom, white: H atom.)

To analyze the reason why Ag@CD NPs can recognize D-ribose and L-ribose, DFT computation was conducted. As is well known, monosaccharides, such as ribose, exist in linear chain form (hereafter abbreviated as linear ribose) or several circular forms in solution, simultaneously; there is an extremely complex dynamic equilibrium between these configurations [27,28,29]. Ribose exists in the β-D-ribofuranose form (hereafter abbreviated as ribofuranose) in the sugar backbones of ribonucleic acids (RNA) and adenosine triphosphate (ATP) because of its special stability. Therefore, in this study, the two most typical configurations considered are linear ribose and ribofuranose. To study the different chiralities of ribose, the four ribose molecules of two chiralities in two configurations were calculated in the DFT analysis. They are named linear L-ribose, linear D-ribose, L-ribofuranose, and D-ribofuranose, respectively; their optimized structures are shown in Figure 6, and the structural parameters are listed in Tables S2–S5 of the Supplementary Materials. The linear ribose contains four hydroxyl groups and one aldehyde group, where three hydroxyl groups are connected to three chiral carbon atoms (C2, C3, and C4). The chiralities of C2, C3, and C4 for L-ribose are *R*, *S*, and *S*, respectively; in contrast, the chiralities of C2, C3, and C4 for D-ribose are *S*, *R*, and *R*, respectively. In the ribofuranose configuration, C1 and C4 are connected by an ether bond to form an oxygen-containing five-membered ring, while C2, C3, and C4 maintain the same chirality as in the chain configuration. The structures of linear L-ribose and linear D-ribose are mirror symmetric; therefore, they have the same energy. In addition, L-ribofuranose and D-ribofuranose are also mirror symmetric and have the same energy.

**Figure 7:**
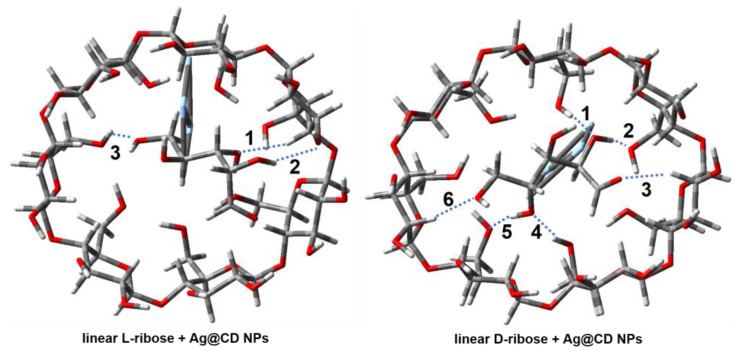

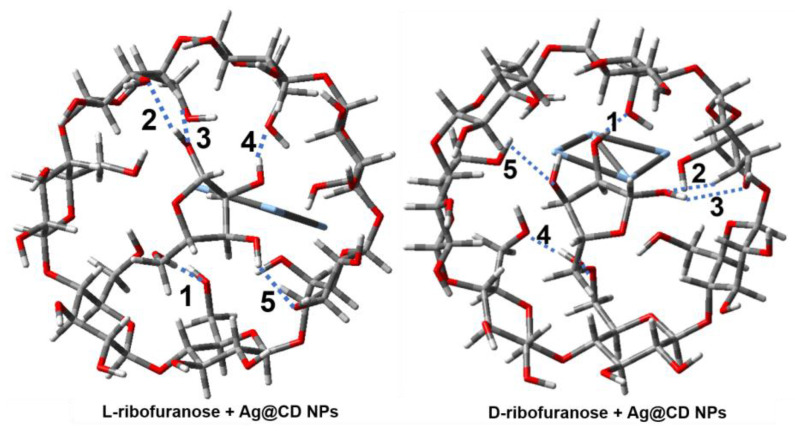
The optimized structure of Ag@CD NP interaction with four ribose molecules under M06-2X/6-31G(d) level of theory for C, O, H atoms and M06-2X/LANL2DZ level of theory for Ag atoms. The blue dashed line represents the intermolecular hydrogen bond between ribose and CD. The color of the atom is set as grey: C atom, red: O atom, white: H atom, silvery: Ag atom.

β-CD is composed of seven D-glucose molecules, and the outer surface of a β-CD appears like a truncated cone; the inner cavity resembles a conical hourglass because of the inward protrusion of the glycosidic oxygens [21]. It is found that many molecules can be embedded into the guest-binding cavity of cyclodextrin to form complexes [30], like quinine [31], polychlorinated biphenyls (PCBs) [32], and thioflavin-T [33]. The glycosidic oxygen and hydroxyl groups of glucose molecules in CD can also provide hydrogen bonds [21]. There are possible interactions between ribose and the cyclodextrin adsorbed on the surface of silver nanoparticles; therefore, these models are set to be composed of D/L-ribose, β-CD, and a silver cluster, as shown in Figure 7. β-CD interacts with the silver cluster at the bottom, and ribose is embedded into the cavity of β-CD. Silver clusters are composed of four silver atoms, which can appropriately describe the interaction between nanoparticles and surface molecules [34].

The optimized structures of the complexes for the four ribose molecules with β-CD are shown in Figure 7, respectively, and the structural parameters are listed in Tables S6–S10 of the Supplementary Materials. The corresponding binding energies for these complexes are listed in Table 1, while the electronic energies and energies corrected by the zero-point energy of molecules and complexes are listed in Table S1 of the Supplementary Materials. Each ribose molecule is embedded into the cavity of β-CD, and ribose interacts with β-CD through several intermolecular hydrogen bonds. The hydrogen bond lengths are listed in Table 2. First, the linear ribose is discussed. There are only three intermolecular hydrogen bonds between the hydroxyl groups of linear L-ribose and the hydroxyl groups of β-CD, whose average bond length is 2.122 Å. However, there are six intermolecular hydrogen bonds between linear D-ribose and β-CD, whose average bond length is only 1.905 Å. Moreover, among them, three hydrogen bonds are shorter than 1.8 Å, which leads to the strong binding energy between linear D-ribose and β-CD. From the top view of β-CD in Figure 7, it can be seen that when linear D-ribose is embedded, β-CD no longer remains round but is obviously distorted into an ellipse due to the strong intermolecular interaction. The binding energy between linear D-ribose and β-CD is −125.877 kJ mol^−1^, much larger than that of linear L-ribose which is only −73.406 kJ mol^−1^. Second, the ribofuranoses are analyzed. There are five intermolecular hydrogen bonds between the hydroxyl groups of L-ribofuranose and β-CD, whose average bond-length is 2.056 Å. There are also five intermolecular hydrogen bonds in the D-ribofuranose and Ag@CD NP complex, and the average bond-length is 2.039 Å. However, there are two very short intermolecular hydrogen bonds (1.679 and 1.830 Å), which would enhance the binding energy of β-CD and D-ribofuranose. As shown in Table 1, the binding energy of D-ribofuranose and Ag@CD NPs is −68.250 kJ mol^−1^, which is about 15 kJ mol^−1^ stronger than that of L-ribofuranose and Ag@CD NPs. When interacting with L-ribofuranose or D-ribofuranose, β-CD maintains a shape close to a circle, as shown in Table 1 and Figure 7. In general, whether in linear or ribofuranose configuration, the binding energy of D-ribose and β-CD is larger than that of L-ribose and β-CD. It is possible that D-ribose has the same chirality as D-glucose on cyclodextrin, and the intermolecular interaction like hydrogen bonds and dipole–dipole effects may be stronger. Compared with the other three molecules, linear D-ribose and β-CD have significantly stronger binding energy. The possible reason is that under the linear configuration, the structure of the D-ribose molecule has a certain flexibility and can fully form more hydrogen bonds with the β-CD with the same chirality by rotating the dihedral angles of the chain.

In this chiral detection strategy, only after adding BR buffer solution with a pH value of 3.2, did the color of the D-ribose mixture change. The reason why acidic conditions are necessary for the chiral recognition can be explained by combining DFT calculations. It is well known that, under acidic conditions, the ring-opening reaction of epoxide is easy to proceed [35,36]. Therefore, the addition of an acidic pH buffer may promote the opening of the oxygen-containing, 5-membered ring of ribofuranose and lead to an increase in the proportion of linear ribose in the dynamic equilibrium of ribose configuration. Since linear D-ribose and β-CD have significantly stronger binding energy, which is 50 kJ mol^−1^ stronger than linear L-ribose, linear D-ribose is more easily and stably embedded into cyclodextrin than ribose in other configurations. From Figure 7 and Table 1, it can be clearly observed that linear D-ribose causes the most severe deformation of β-CD. This may change the surface properties, such as the charge of the Ag@CD NPs, leading to the aggregation and precipitation of nanoparticles, subsequently changing the color of colloids and surface plasmon absorption. Therefore, D-ribose and L-ribose can be distinguished by both visual and SERS assessments. Since cyclodextrins have different interactions with chiral enantiomers, cyclodextrins will also have important applications in chiral separation science, such as capillary electrophoresis, gas chromatography, and liquid chromatography [37].

## 3. Conclusions

This study demonstrates that the chiral recognition of D/L-ribose can be achieved through both visual and SERS assessments. The enantiomeric ribose can be identified due to the optical signals induced by Ag@CDNP aggregation. The color of mixture of Ag@CD NPs, D-ribose, and acidic BR turns purple–red and gray–green over time, while the color of L-ribose stays yellow. Particularly, the SERS method provides the chiral discrimination of D/L-ribose with higher sensitivity. Based on DFT analysis, the mechanism of chiral recognition is inferred. Under acidic conditions, it is easy for ribose to open its oxygen-containing ring to form linear ribose. Linear D-ribose and cyclodextrin have the same chirality, and linear ribose is flexible, thus forming multiple strong hydrogen bonds between them. Strong binding changes the structural and electronic properties of cyclodextrin, which leads to aggregation of silver nanoparticles and changes in optical properties. Since the traditional chiral recognition scheme of ribose requires chromatography and other methods, which are very time-consuming and expensive, this study provides more convenient chiral recognition approaches and may facilitate the relevant research of ribose chirality.

## 4. Materials and Methods

### 4.1. Reagents and Materials

D-ribose, L-ribose, AgNO_3_, H_3_BO_3_, CH_3_COOH, H_3_PO_4_, NaOH, HCl were purchased from Sinopharm Chemical Reagent Co., Ltd. (Shanghai, China). β-cyclodextrin (β-CD, C_42_H_70_O_35_, molecular weight is 1134.99) composed of D-glucose was purchased from Sangon Biotechnology Inc. (Shanghai, China). High-purity water was used in this study. All the chemicals were used as received without further purification.

### 4.2. Preparation of Ag@CD NPs (β-cyclodextrin Coated Ag Nanoparticles)

The Ag@CD NPs were prepared via the reduction of AgNO_3_ by β-CD under alkaline conditions. A quantity of 80 mg of β-CD was dissolved in 10 mL of H_2_O at 80 °C under water bath heating conditions. After that, 50 μL of 1 M NaOH was added, and then, 300 μL of 20 mM AgNO3 was added and reacted for 10 min. The solution’s color changed from colorless to yellow. After this solution had cooled down to ambient temperature, the Ag@CD NPs were washed with ultrapure water three times by centrifugation at about 8000 rpm to remove excess β-CD; then, Ag@CD NPs was dissolved in 10 mL of ultrapure water. The silver colloids were yellow and showed a visible absorption band at 400 nm. Transmission electron microscope (TEM) imaging was performed on a Talos F200X microscope operated at an accelerating voltage of 200 kV. The silver colloid was dropwise added to a dedicated copper mesh and then vacuum dried for TEM detection. Silver nanoparticles exist as spheres with a diameter of about 20 nm.

### 4.3. Experimental Processing of Chiral Recognition

A typical chiral recognition was realized by the following procedure. In the centrifuge tube, 200 μL of Ag@CD NPs, 200 μL of L-ribose or D-ribose at the appropriate concentration, and 20 μL of Britton–Robinson (BR) buffer solution at pH = 3.2 were added successively. The mixed solution was placed at room temperature for 30 min. The color changes of the sol were monitored and photographed, and then, the UV–Vis and SERS spectra were measured.

### 4.4. UV–vis Absorption Spectra Measurements

The UV–vis absorption spectra were recorded on a U-3900H spectrophotometer (Hitachi, Japan) at room temperature. The pH measurements were carried out on a model PB-10 digital pH-meter (Sartorius Scientific Instruments Co., Ltd., Beijing, China). The value of absorbance (A) of the UV–vis absorption spectra was the average of five repeats and then used in this study without baseline correction, curve fitting, or normalization.

### 4.5. SERS Measurements

The sample was dropped onto a quartz plate for SERS measurement. All the Raman spectra were recorded using an Oceanhood RMS 1000 portable Raman spectrometer. The SERS spectra were measured in the range of 400–2000 cm^−1^ using a 785 nm diode laser, and the laser power at the sample was ca. 50 mW for 20 s. The spectra were measured with at least three repeats for average and then used in this work without baseline correction, curve fitting, or normalization.

### 4.6. DFT Calculation

The DFT calculations were carried out using the D. 01 version of Gaussian 09 software [38]. In the calculation, the M06-2X functional was applied, and 6-31G(d) basis sets were used for C, O, H atoms, while the pseudopotential basis sets Lanl2DZ were used for the Ag atoms [39,40]. The M06-2X (hybrid meta-generalized gradient approximation exchange−correlation functional) is a high-nonlocality functional with double the amount of nonlocal exchange (2X) for nonmetals. The M06-2X functional is based on a different approach to correct the dispersion effect and has been optimized to give good performance for most kinds of properties, including noncovalent interactions [41,42,43]. Therefore, the M06-2X functional is suitable for structure optimization and energy calculation of the interactions between biomolecules, such as stacked base pairs [44] and amino acids [45]. The tight convergence criterion of Gaussian 09 was used in structure optimization, and the ultrafine integration grid was used in the numerical integration of the structure optimization and vibrational frequency calculation. The geometries were fully optimized without any constraint on the geometry, and the optimized structures had no imaginary frequencies. The calculations of the harmonic vibrational wavenumbers and relative Raman activities were carried out at the same level of theory using the same basis sets. The vibrational frequency assignments were made based on the results of the Gaussview program 5.0.8 version, and the potential energy distribution (PED) matrix was expressed in terms of a combination of local symmetry and internal coordinates [46]. The binding energy *E*(bind) was calculated according to the following formula: *E*(bind) = *E*(AB) − *E*(A) − *E*(B), where *E*(AB) is the energy of complex AB, *E*(A), and *E*(B) are the energies of fragment A and fragment B, respectively. It should be noted that the energy of every structure, like *E*(AB), *E*(A), or *E*(B), has been corrected with the zero point energy according to the following formula: *E*(corrected energy) = *E*(electronic energy) + *E*(zero point energy correction).

## Figures and Tables

**Table 1:** The binding energies (in kJ mol^−1^) of four kinds of ribose molecules interacting with β-CD, and the dimensions (in Å × Å) of β-CD on the top view.

Complex	E(Bind) (in kJ mol^−1^)	Size ^a^ (in a Å × b Å)
linear L-ribose and Ag@CD	−73.406	14.2 × 13.4
linear D-ribose and Ag@CD	−125.877	15.4 × 12.8
L-ribofuranose and Ag@CD	−53.342	14.1 × 13.4
D-ribofuranose and Ag@CD	−68.250	14.3 × 13.5

^a^ The molecular size measurement on the top view: first, find the two farthest atoms of β-CD and measure their distance a; then, find the two farthest atoms perpendicular to two previous atoms and measure their distance b.

**Table 2:** The numbers and bond lengths (in Å) of intermolecular hydrogen bonds between ribose and Ag@CD NP complexes.

Complex	1 ^a^	2	3	4	5	6	Avg. ^b^
linear L-ribose and Ag@CD	2.198	2.098	2.069				2.122
linear D-ribose and Ag@CD	1.774	1.644	2.269	1.949	1.738	2.055	1.905
L-ribofuranose and Ag@CD	1.989	2.142	2.121	1.923	2.106		2.056
D-ribofuranose and Ag@CD	1.679	2.169	2.288	1.830	2.228		2.039

^a^ The sort number of intermolecular hydrogen bonds (hydrogen bond length less than 2.300 Å has been counted). ^b^ The average bond length of the hydrogen bonds.

## Data Availability

All the data are embedded in the manuscript.

## Supplementary Materials

The following supporting information can be downloaded at https://www.mdpi.com/article/10.3390/molecules28186480/s1: Table S1. The energies (in Hartree) of ribose molecules and complexes under M06-2X/6-31G(d) level of theory for C, O, H atoms and M06-2X/LANL2DZ level of theory for Ag atoms. Table S2. The Cartesian coordinates (in Å) of atoms in the optimized structure of linear L-ribose. Table S3. The Cartesian coordinates (in Å) of atoms in the optimized structure of linear D-ribose. Table S4. The Cartesian coordinates (in Å) of atoms in the optimized structure of L-ribofuranose. Table S5. The Cartesian coordinates (in Å) of atoms in the optimized structure of D-ribofuranose. Table S6. The Cartesian coordinates (in Å) of atoms in the optimized structure of β-cyclodextrin on silver cluster (Ag@CD). Table S7. The Cartesian coordinates (in Å) of atoms in the optimized structure of the complex of linear L-ribose and Ag@CD. Table S8. The Cartesian coordinates (in Å) of atoms in the optimized structure of the complex of linear D-ribose and Ag@CD. Table S9. The Cartesian coordinates (in Å) of atoms in the optimized structure of the complex of L-ribofuranose and Ag@CD. Table S10. The Cartesian coordinates (in Å) of atoms in the optimized structure of the complex of D-ribofuranose and Ag@CD. Figure S1. Up: The UV-Vis spectra of beta-CD@Ag nanoparticles before (in black) and after (in red) adding BR. Down: The photographs of beta-CD@Ag nanoparticles before (left) and after (right) adding BR. The UV-Vis spectrum and the color of beta-CD@Ag nanoparticles is nearly the same before and after adding BR. Figure S2. The photographs of the mixture of 200μL Ag@CD NPs (abbreviated as AgNPs in the figures), 200 μL 0.1M L-glucose or D-glucose or L-Mannose or D-Mannose, 30 min after adding 20 μL BR of pH = 3.2. Figure S3. (a) R value minus B value of the photos of Ag@CD NPs responding to a series of different concentrations of D-ribose (from left to right: 0.1, 0.01, 0.001, 10^−4^, 10^−5^, 10^−6^ M) after adding BR for 30 min. (b) The relative data of the photos of Ag@CD NPs of different concentrations of D-ribose after adding BR for 30 min. a R, G, B values of the photos of D-ribose and Ag@CD NPs mixture using image software. R values are used for the fitting. Figure S4. SERS spectra of Ag@CD NPs before (in black) and after (in red) adding BR for 30 min.

## Sample Availability

Samples of the compounds are available from the authors.
