# Supplementary material for: Chiral Recognition of D/L-Ribose by Visual and SERS Assessments
Source: Molecules. 2023 Sep 7;28(18):6480. doi: 10.3390/molecules28186480 (PMC10537478; doi:10.3390/molecules28186480)
Supplement: Supplementary file 1 [file molecules-28-06480-s001.zip › molecules-2465718-supplementary.pdf]

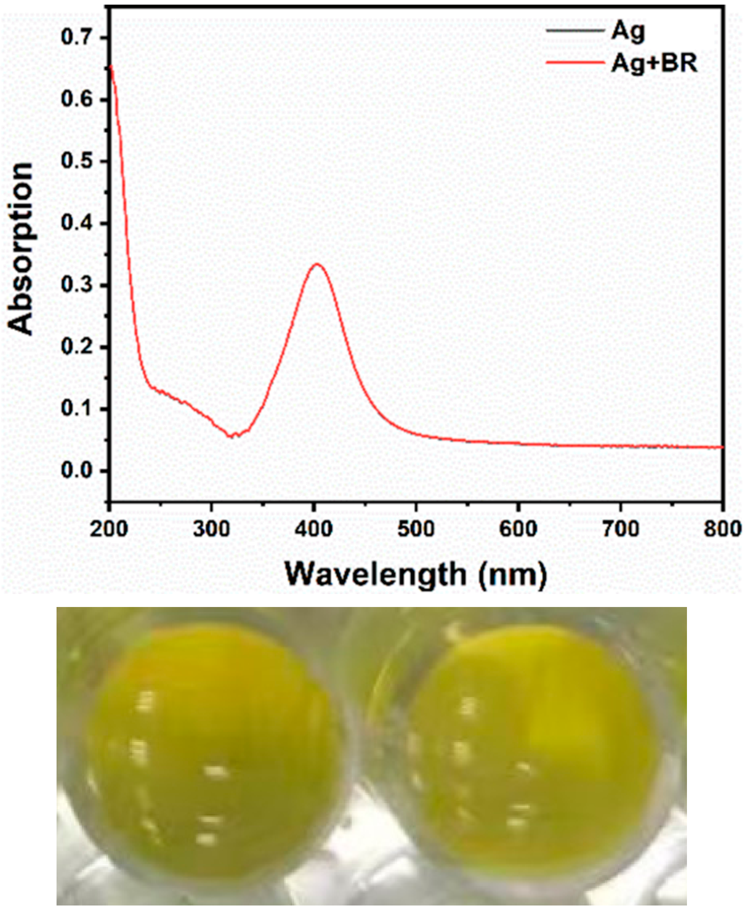

Figure S1. Up: The UV-Vis spectra of beta-CD@Ag nanoparticles before (in black) and after (in red) adding BR. Down: The photographs of beta-CD@Ag nanoparticles before (left) and after (right) adding BR. The UV-Vis spectrum and the color of beta-CD@Ag nanoparticles is nearly the same before and after adding BR.

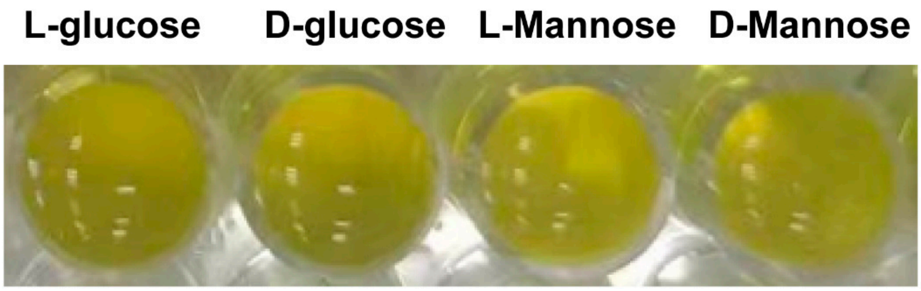

Figure S2. The photographs of the mixture of 200μL Ag@CD NPs (abbreviated as AgNPs in the figures), 200 μL 0.1M L-glucose or D-glucose or L-Mannose or D-Mannose, 30 min after adding 20 μL BR of pH=3.2.

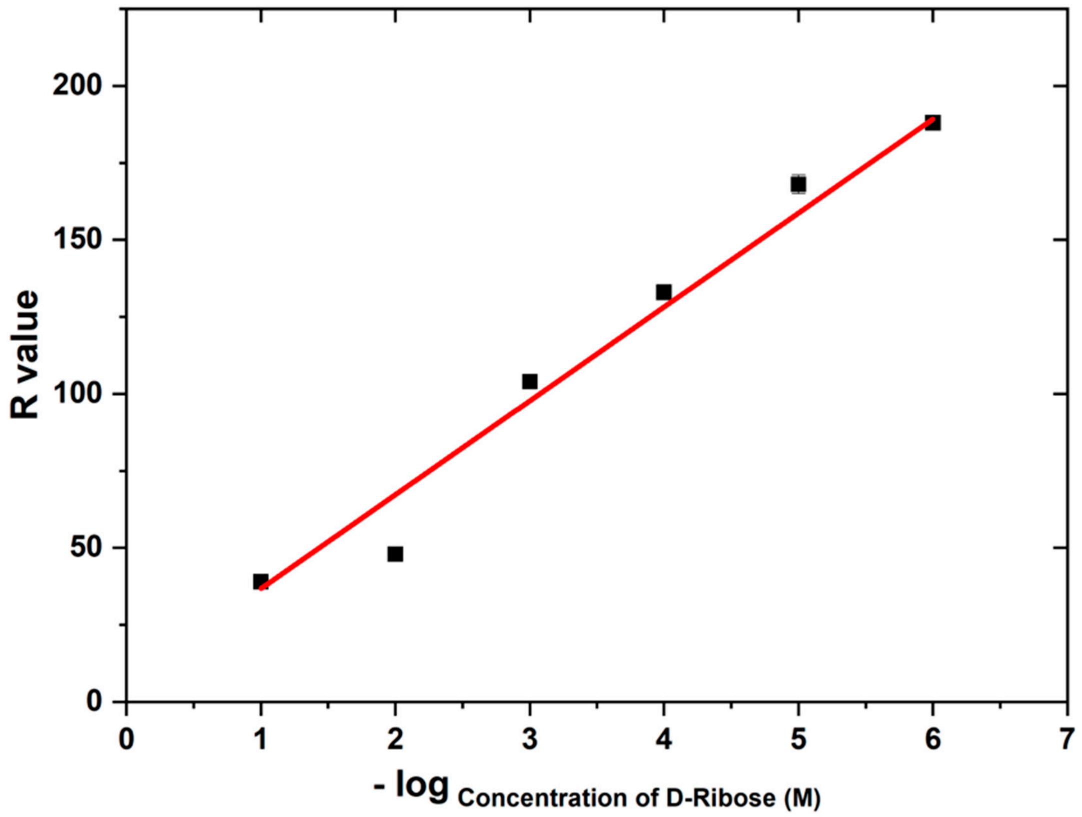

Concentration (M)	R [a]	G [a]	B [a]
0.1	39	35	38
0.01	48	42	38
0.001	104	92	37
10^{-4}	133	119	39
10^{-5}	168	143	37
10^{-6}	188	158	40

Figure S3. (a) R value minus B value of the photos of Ag@CD NPs responding to a series of different concentrations of D-ribose (from left to right: 0.1, 0.01, 0.001, 10^{-4}, 10^{-5}, 10^{-6} M) after adding BR for 30 minutes. (b) The relative data of the photos of Ag@CD NPs of different concentrations of D-ribose after adding BR for 30 minutes.

a R, G, B values of the photos of D-ribose and Ag@CD NPs mixture using image software. R values are used for the fitting.

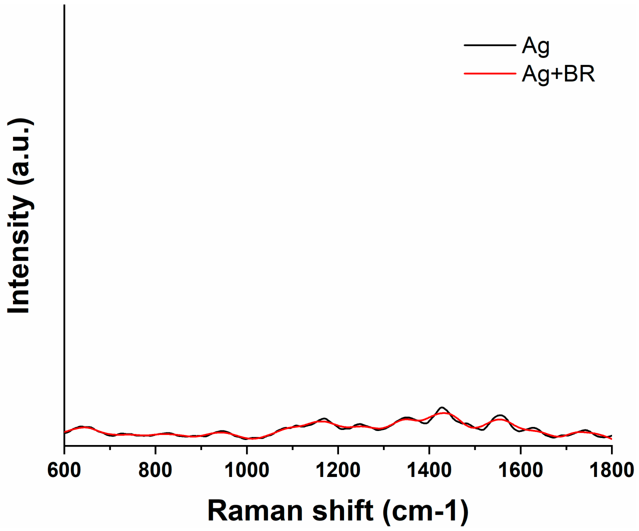

Figure S4. SERS spectra of Ag@CD NPs before (in black) and after (in red) adding BR for 30 mins.

Table S1. The energies (in Hartree) of ribose molecules and complexes under M06-2X/6-31G(d) level of theory for C, O, H atoms and M06-2X/LANL2DZ level of theory for Ag atoms.

	linear L-ribose	linear D-ribose	L-ribofuranose	D-ribofuranose	Ag@CD	linear L-ribose and Ag@CD	linear D-ribose and Ag@CD	L-ribofuranose and Ag@CD	D-ribofuranose and Ag@CD
E(electronic energy)	-572.3879	-572.3879	-572.4219	-572.4219	-4856.5101	-5428.9283	-5428.9481	-5428.9573	-5428.9624
E(corrected energy) a	-572.2228	-572.2228	-572.2552	-572.2552	-4855.2612	-5427.5119	-5427.5319	-5427.5367	-5427.5424

[a] E(corrected energy) is corrected with the zero point energy according to the following formula, *E*(corrected energy) = *E*(electronic energy) + *E*(zero point energy correction).

Table S2. The Cartesian coordinates (in Å) of atoms in the optimized structure of linear L-ribose.

O	0.54696600	0.95304900	-1.38108000
O	-1.27165300	-0.35085400	1.54701500
O	2.56401200	1.06684900	0.54484900
O	-3.35309900	-0.36589600	-0.29257500
O	1.52089100	-1.64230300	-0.91279700
C	0.24207100	0.72202000	-0.01699000
C	-1.00427300	-0.14186500	0.16209900
C	1.47849200	0.17108300	0.69184100
C	-2.23514700	0.45976500	-0.50155700
C	1.95738900	-1.15258000	0.10490700
H	0.04517200	1.71571300	0.41131800
H	-0.83508400	-1.14150100	-0.25069300
H	1.29007200	0.04690900	1.76279000
H	-2.08260800	0.56234000	-1.57831800
H	-2.39583300	1.47415100	-0.09016600
H	0.69015300	0.07486900	-1.77604100
H	2.81618100	-1.61166500	0.63025500
H	-1.42494100	0.51969800	1.94957800
H	2.50464900	1.38688500	-0.37288500
H	-3.29588200	-0.66468400	0.62905900

Table S3. The Cartesian coordinates (in Å) of atoms in the optimized structure of linear D-ribose.

O	-0.34288300	1.58241800	-0.78067300
O	1.14202300	-1.22524500	0.84406900
O	-2.41256800	0.87920300	0.76190500
O	3.46664700	-0.32747300	-0.28900600
O	-2.75346800	-1.07842000	-0.94711400
C	-0.08452400	0.74140600	0.31589700
C	1.07973500	-0.15556200	-0.07435900
C	-1.35802300	-0.04685000	0.67234700
C	2.40460600	0.59286100	-0.07757600
C	-1.69332500	-1.09843300	-0.36618600
H	0.18971100	1.32689300	1.20867000
H	0.88528400	-0.52657700	-1.09724600
H	-1.19416800	-0.56961500	1.62590000
H	2.57460800	1.02608800	0.91303500
H	2.39443800	1.39966500	-0.81925900
H	-0.94556100	-1.89128100	-0.53704500
H	-1.20187600	1.99494000	-0.58693800
H	2.04628000	-1.57642100	0.77566100
H	-3.13341100	0.51387400	0.21527500
H	3.49586900	-0.54196400	-1.23223500

Table S4. The Cartesian coordinates (in Å) of atoms in the optimized structure of L-ribofuranose.

C	-2.32714800	0.08181900	0.00813400
H	-2.94299600	-0.51550900	-0.67651300
H	-2.87541100	1.00229900	0.24792200
C	-1.04072000	0.45798200	-0.69442300
H	-1.29630300	1.08106400	-1.55980400
O	-0.37353100	-0.72858600	-1.16056100
C	0.91898700	-0.83230600	-0.58299700
H	1.67501700	-0.49174600	-1.30207500
C	-0.02295000	1.20346300	0.18559700
H	-0.50691300	1.72577900	1.01840200
C	0.88274800	0.07159100	0.64399800
H	0.38111800	-0.48156400	1.44637300
O	2.15459600	0.53153700	1.04462200
H	2.63735400	-0.23863900	1.38559400
O	1.17833800	-2.14955200	-0.18853300
H	1.44815700	-2.65370400	-0.97250900
O	-2.01849500	-0.65380000	1.18182900
H	-2.85496500	-0.86083400	1.62570200
O	0.77003700	2.09413600	-0.58388200
H	0.18187100	2.78768900	-0.92273700

Table S5. The Cartesian coordinates (in Å) of atoms in the optimized structure of D-ribofuranose.

C	2.34515300	0.04699100	-0.05225100
H	2.88046600	-0.70657600	-0.64523700
H	2.95845500	0.96000400	-0.01078000
C	1.04073700	0.36573600	-0.75440000
H	1.25061500	0.91927900	-1.67749500
O	0.36990200	-0.85632500	-1.08075200
C	-0.92392600	-0.86116300	-0.52052200
H	-1.65139600	-0.49875700	-1.26466800
C	0.06551000	1.18540800	0.10296000
H	0.58028900	1.74663500	0.89317100
C	-0.85244100	0.10165300	0.65613900
H	-0.37158500	-0.41667200	1.49352900
O	-2.09695600	0.66542600	1.00710000
H	-2.69813200	-0.06140400	1.22491300
O	-1.27531400	-2.12610600	-0.05781600
H	-1.34348000	-2.71882800	-0.81984600
O	2.04446100	-0.42882400	1.24489100
H	2.84915700	-0.78760700	1.63993600
O	-0.66447200	2.04019000	-0.75121800
H	-1.52554600	2.17728700	-0.32270900

Table S6. The Cartesian coordinates (in Å) of atoms in the optimized structure of β-cyclodextrin on silver cluster (Ag@CD).

C	6.39552400	1.19314200	-1.05674700
C	6.18343400	-0.29697200	-1.25307600
C	6.32688800	-0.98436100	0.10365400
C	5.46866400	0.92239700	1.26029600
C	5.41650600	1.74337600	-0.03549900
H	5.16145100	-0.45539900	-1.63037200
H	7.41876300	1.35888200	-0.68269200
H	6.43077600	1.12548900	1.75858800
H	4.39871600	1.68749500	-0.43819300
H	7.33695500	-0.80622900	0.50100500
O	6.21899600	1.86532300	-2.29082200
H	6.69663600	1.33028000	-2.94865900
O	7.13860500	-0.73075000	-2.18383500
H	6.85486200	-1.62108400	-2.47121000
C	4.32482200	1.23381700	2.21602700
H	4.43639000	0.59431100	3.09912100
H	4.36408100	2.28042300	2.52139200
O	3.08471500	0.90937600	1.61120300
H	2.61109100	1.70882400	1.29306100
O	5.34908800	-0.46370800	0.97993300
O	5.79439300	3.07954900	0.26388900
O	6.18856700	-2.36339200	0.06201500
C	4.92914600	-2.87103900	-0.35698600
C	5.15247700	-3.77271800	-1.55716900
C	4.31927400	-3.67115600	0.80022400
H	4.23331700	-2.06736800	-0.62450600
C	3.86943100	-4.49681700	-1.92299700
H	5.92062000	-4.51809300	-1.29499100
O	5.58919400	-2.99904400	-2.66090400
H	5.08488100	-4.37226800	1.17295700
C	3.83325500	-2.79645400	1.94881800
O	3.17516300	-4.38022800	0.36249600
C	3.38396100	-5.27834200	-0.70242000
H	3.11565900	-3.74098300	-2.19046800
O	4.16071400	-5.32627800	-3.01649300
H	5.48635000	-3.57562400	-3.43827500
H	3.42384000	-3.45274400	2.72390200
H	4.67051000	-2.23249800	2.36474600
O	2.78993200	-1.94306500	1.52038300
H	4.13324200	-6.03792700	-0.43354900
H	3.29851700	-5.60735900	-3.38198300
H	3.11906000	-1.03926900	1.34890500
O	2.20643700	-5.98348200	-0.90887900
C	1.05202600	-5.22660400	-1.25113000
C	0.52340300	-5.76325100	-2.56891600
C	0.00646700	-5.38610600	-0.13753600
H	1.27586700	-4.15865400	-1.34467100
C	-0.82825200	-5.15627000	-2.89347400
H	0.40961600	-6.85575300	-2.47993600
O	1.43943700	-5.45841300	-3.60658400
H	-0.07118700	-6.45444200	0.12072400
C	0.33909000	-4.56777300	1.10228600
O	-1.25645500	-4.89142400	-0.55893900
C	-1.77914600	-5.45777000	-1.73721800

H	-0.70340400	-4.06646200	-2.98336200
O	-1.25415900	-5.71767300	-4.10691100
H	0.95461300	-5.61793900	-4.43515100
H	-0.44716400	-4.73998400	1.84851600
H	1.29796100	-4.88956300	1.51021800
O	0.35419100	-3.20147100	0.73670100
H	-1.89679400	-6.54641300	-1.63197300
O	-3.06645000	-4.96790500	-1.91710500
H	-1.99803300	-5.16491400	-4.41715400
H	1.17780400	-2.75888100	1.03101800
C	-3.21010300	-3.55947600	-2.03496100
C	-3.90964100	-3.27540100	-3.35359600
C	-4.04795000	-3.05415000	-0.85173300
H	-2.24199400	-3.04611900	-2.01203700
C	-4.32802100	-1.81910900	-3.45116300
H	-4.81347500	-3.90355600	-3.40638100
O	-3.03999700	-3.59229000	-4.42595900
H	-4.93530400	-3.69916100	-0.75191200
C	-3.28738000	-3.03150900	0.46737400
O	-4.45496800	-1.70816800	-1.07355500
C	-5.21365400	-1.50114300	-2.24866400
H	-3.42293000	-1.19327000	-3.40808800
O	-4.99468700	-1.66344600	-4.67471800
H	-3.45023100	-3.20283200	-5.21780000
H	-3.95795900	-2.64898200	1.24672100
H	-2.96916900	-4.04112000	0.73043000
O	-2.18196600	-2.14396000	0.36899500
H	-6.09670600	-2.15574900	-2.25490500
H	-5.07665300	-0.70092600	-4.82421500
H	-1.32197100	-2.62415300	0.37459700
O	-5.71930800	-0.20898500	-2.23129900
C	-4.77852900	0.84957500	-2.14347700
C	-4.97775300	1.78045900	-3.32543400
C	-5.00385200	1.60134500	-0.82393900
H	-3.74732500	0.47821500	-2.15908000
C	-4.10842900	3.01599500	-3.16935100
H	-6.03542200	2.08720200	-3.36006800
O	-4.63062800	1.10482700	-4.52050500
H	-6.07318000	1.85573600	-0.74448400
C	-4.57515900	0.80321900	0.39854700
O	-4.21509300	2.77941000	-0.79200600
C	-4.45611000	3.69136000	-1.84313700
H	-3.05606700	2.69382200	-3.14206700
O	-4.35287000	3.83988100	-4.27724600
H	-4.54580200	1.79880600	-5.19754100
H	-4.68802900	1.42809400	1.28934100
H	-5.18833700	-0.09295200	0.51387000
O	-3.19649900	0.47168600	0.29507900
H	-5.51146100	4.00089300	-1.85328500
O	-3.73630800	4.84776900	-1.58776300
H	-3.63244200	4.50090200	-4.28789700
H	-3.11037700	-0.39010900	-0.15960100
C	-2.32779200	4.71124700	-1.45181600
C	-1.66728900	5.61871200	-2.47368800
C	-1.93196500	5.12831200	-0.02999100
H	-1.99977100	3.67731500	-1.61001400
C	-0.16826600	5.68148700	-2.24444000
H	-2.08671500	6.63147200	-2.36094100

O	-1.92807700	5.13280600	-3.77920900
H	-2.41365100	6.09603600	0.18913600
C	-2.33040300	4.11425400	1.03605100
O	-0.52527800	5.24540600	0.07699200
C	0.08117400	6.15322700	-0.81220000
H	0.23955500	4.66661600	-2.36640200
O	0.36006900	6.55685600	-3.20513400
H	-1.30353200	5.60234800	-4.35954400
H	-2.00902900	4.50463600	2.00730200
H	-3.41528100	3.99333700	1.04435300
O	-1.66749800	2.88110200	0.83318600
H	-0.33269700	7.16466100	-0.68356900
H	1.32880500	6.42487800	-3.19185000
H	-2.26063100	2.22862400	0.41554200
O	1.42209100	6.26202900	-0.47123100
C	2.20262300	5.07467700	-0.52816300
C	3.37509800	5.34251100	-1.45438000
C	2.68340300	4.72974300	0.88989400
H	1.62408100	4.22198500	-0.89836200
C	4.38558100	4.21301100	-1.38946200
H	3.86684100	6.27531400	-1.13427100
O	2.90210100	5.48013700	-2.78330300
H	3.05828000	5.64893000	1.36754600
C	1.59052800	4.09886900	1.74183900
O	3.71007400	3.74753500	0.85116800
C	4.83440200	4.06245100	0.06208700
H	3.88971800	3.28442800	-1.71128500
O	5.43940500	4.54962200	-2.25214900
H	3.69350000	5.42845900	-3.34715100
H	2.01581200	3.86377400	2.72710600
H	0.76574100	4.80121900	1.86542500
O	1.17703600	2.90472300	1.10579900
H	5.30242800	4.99783000	0.40259500
H	5.96403500	3.73405100	-2.37361300
H	0.19932300	2.85879700	1.04062600
Ag	-1.87266200	-0.43878400	2.22032100
Ag	-1.13560600	-0.20908100	4.87700600
Ag	-3.96811600	-0.83734400	4.31495300
Ag	0.95145600	0.19476800	2.96309100

Table S7. The Cartesian coordinates (in Å) of atoms in the optimized structure of the complex of linear L-ribose and Ag@CD.

C	0.58726100	-6.14025300	-0.34749800
C	-0.85727600	-5.67918300	-0.45362900
C	-1.48342200	-5.80091200	0.93723900
C	0.58393100	-5.18439400	1.99075200
C	1.34145000	-5.29144700	0.65879200
H	-0.84107800	-4.61525200	-0.73693400
H	0.60821400	-7.19106100	-0.01851900
H	0.71617900	-6.13178200	2.53823200
H	1.45845200	-4.28276900	0.24673600
H	-1.39084800	-6.83638400	1.29754300
O	1.21300900	-6.01833000	-1.61563800
H	0.56682300	-6.37395100	-2.25202700
O	-1.46807600	-6.46964100	-1.43025300
H	-2.36777600	-6.11655200	-1.60021300
C	1.07266800	-4.01893700	2.84209700
H	0.43932100	-3.95833000	3.73367800
H	2.10888300	-4.17420900	3.14206000
O	0.92553300	-2.80402200	2.11746300
H	1.79631900	-2.42771500	1.85224600
O	-0.78993600	-4.91878000	1.79164400
O	2.60055100	-5.90063900	0.91469000
O	-2.83526300	-5.51128000	1.02280700
C	-3.31484900	-4.32267200	0.41518200
C	-4.53444000	-4.70119100	-0.40817400
C	-3.66362400	-3.27090700	1.48423800
H	-2.57769400	-3.88385400	-0.25940500
C	-5.13752600	-3.43305400	-0.98401200
H	-5.27456100	-5.19151400	0.24562700
O	-4.15499200	-5.59360000	-1.43290400
H	-4.24407100	-3.75677200	2.28366700
C	-2.44043300	-2.58952500	2.08218600
O	-4.42675400	-2.20235100	0.92361600
C	-5.58097700	-2.56786100	0.18936900
H	-4.35089100	-2.89372100	-1.53165000
O	-6.19246700	-3.79637100	-1.83202700
H	-4.77004900	-5.43360900	-2.16939100
H	-2.77739600	-1.72815900	2.67291500
H	-1.91667300	-3.28514300	2.73683700
O	-1.57514800	-2.14490100	1.03814000
H	-6.28653400	-3.12079100	0.82576400
H	-6.38121700	-3.01005600	-2.37673900
H	-0.66013900	-2.38034800	1.30465500
O	-6.25133300	-1.40873400	-0.18603600
C	-5.44871100	-0.40670900	-0.78966000
C	-5.91433900	-0.11666200	-2.20156400
C	-5.56020100	0.85876900	0.06929800
H	-4.39889700	-0.70824300	-0.84275800
C	-5.07638800	1.02606200	-2.75036900
H	-6.97873800	0.16574300	-2.19995300
O	-5.73020700	-1.26969700	-3.01071100
H	-6.62951100	1.10469900	0.18266400
C	-4.93736200	0.69183900	1.44870000
O	-4.86848500	1.93521900	-0.53557500
C	-5.25719800	2.25065500	-1.85235500
H	-4.02408800	0.70860300	-2.71953800

O	-5.48887500	1.26068000	-4.07046100
H	-5.70598900	-0.94419200	-3.93055600
H	-5.08268700	1.61379700	2.01792900
H	-5.42418400	-0.13444300	1.97502800
O	-3.53882800	0.49242500	1.36555300
H	-6.30651200	2.57907500	-1.88495000
O	-4.51677500	3.35490500	-2.25561200
H	-4.75678900	1.74632300	-4.49610200
H	-3.37815200	-0.41271200	1.04706100
C	-3.10154100	3.22109900	-2.25351800
C	-2.62316500	3.53052500	-3.66222000
C	-2.52324800	4.21369900	-1.23378700
H	-2.78693400	2.21139900	-1.95951900
C	-1.12514000	3.76700300	-3.73363000
H	-3.12856300	4.45004300	-3.99740700
O	-2.96859400	2.45060900	-4.51546600
H	-3.02724400	5.18419400	-1.36151800
C	-2.66147900	3.74193100	0.20632900
O	-1.12147100	4.37485000	-1.43582000
C	-0.75920700	4.84680800	-2.71710000
H	-0.61101500	2.83182600	-3.45223600
O	-0.84042300	4.12904900	-5.05601200
H	-2.52748800	2.63597000	-5.36281800
H	-2.18981900	4.48462000	0.86183800
H	-3.71611100	3.65137500	0.47016400
O	-1.97624300	2.50813200	0.35946700
H	-1.30466000	5.77254100	-2.94986900
H	0.13265600	4.10326700	-5.15052400
H	-2.61453100	1.77933600	0.54667000
O	0.57763300	5.20918700	-2.69795600
C	1.53739500	4.20481200	-2.39539800
C	2.49539100	4.11479100	-3.56633100
C	2.29334500	4.60141400	-1.11747000
H	1.08060600	3.22325100	-2.22678800
C	3.70090500	3.25869500	-3.23818200
H	2.83807100	5.13193200	-3.81193500
O	1.82843100	3.54687900	-4.68535900
H	2.66862500	5.63122900	-1.24310000
C	1.42874700	4.54913400	0.13583300
O	3.38535600	3.71781000	-0.91383600
C	4.34645900	3.76522100	-1.94956100
H	3.34517000	2.23047600	-3.07257000
O	4.55225400	3.33417100	-4.34933900
H	2.53139400	3.36836700	-5.33516200
H	2.07139300	4.69670300	1.00792300
H	0.68534600	5.35225300	0.10177900
O	0.80438300	3.28749200	0.28023000
H	4.70282400	4.79563100	-2.09526700
O	5.46470700	3.04414800	-1.55798500
H	5.19680600	2.60513600	-4.25233800
H	-0.02109000	3.30813400	-0.24052700
C	5.27502400	1.66353200	-1.28776200
C	6.15936800	0.85573100	-2.21970500
C	5.67161400	1.39060400	0.16791800
H	4.22731100	1.36609400	-1.41229000
C	6.09710900	-0.61240200	-1.85128000
H	7.19539800	1.21570500	-2.11548000
O	5.73065500	1.00347100	-3.56199500

H	6.68249800	1.80109500	0.33000100
C	4.71236200	2.01600200	1.17593800
O	5.65821600	-0.00257700	0.42439800
C	6.50288100	-0.78597400	-0.38983900
H	5.05559000	-0.94448300	-1.97606800
O	6.94615500	-1.29777300	-2.73368700
H	6.16937000	0.28466400	-4.05106600
H	5.09687000	1.81846800	2.18079500
H	4.67402900	3.09703500	1.01698100
O	3.42782800	1.43488700	1.11809100
H	7.55485000	-0.49147500	-0.26199800
H	6.68467300	-2.23806300	-2.68089100
H	2.90598300	1.79014600	0.37110800
O	6.43099300	-2.10172600	0.05005900
C	5.14800900	-2.71393300	0.01184300
C	5.21836600	-3.94353600	-0.87586800
C	4.75509000	-3.08839800	1.44751000
H	4.38309900	-2.02889500	-0.36872400
C	3.92210300	-4.72615900	-0.77474800
H	6.05047300	-4.58063100	-0.53522800
O	5.43102600	-3.55566900	-2.22193100
H	5.61708000	-3.57318300	1.93255800
C	4.31146500	-1.87024300	2.24850200
O	3.63150600	-3.95663800	1.46471900
C	3.72942500	-5.12759100	0.68504700
H	3.09391100	-4.06649200	-1.07717800
O	4.02486800	-5.82955800	-1.63434500
H	5.20683800	-4.33982100	-2.75363600
H	4.03834700	-2.20167100	3.25906400
H	5.13077200	-1.15326800	2.31540100
O	3.17785900	-1.31151100	1.61339500
H	4.57672200	-5.74791600	1.01237600
H	3.11717000	-6.17692800	-1.74079500
H	3.29538900	-0.35005100	1.43002300
Ag	-0.28867000	1.71580800	4.95970100
Ag	-0.31747000	2.32501800	2.25653000
Ag	-0.97536900	4.47038800	4.22303900
Ag	0.41705000	-0.41142100	3.18278800
O	-2.24399800	-0.65057500	-1.27329600
O	0.84707400	-0.36232800	-3.25118600
O	-3.07940400	-1.44442500	-3.89456500
O	1.81056900	0.93705800	-1.16278900
O	-2.31222100	-3.72127900	-2.59949800
C	-1.41969900	-0.50542600	-2.40714700
C	0.06538500	-0.44000400	-2.05911700
C	-1.75503000	-1.56947600	-3.47811300
C	0.40923700	0.74710200	-1.16288500
C	-1.44628000	-2.95136900	-2.94181000
H	-1.70787200	0.45273600	-2.87000600
H	0.39371900	-1.36663900	-1.56905400
H	-1.11507900	-1.39181800	-4.34897100
H	0.06908800	0.58855700	-0.13314300
H	-0.11298200	1.64189900	-1.54422900
H	-1.87204800	-1.26175700	-0.59867200
H	-0.37507300	-3.22366000	-2.84033600
H	0.57016700	0.43206900	-3.74113500
H	-3.67812000	-1.68060300	-3.16378900
H	2.13262200	0.53248800	-1.98721700

Table S8. The Cartesian coordinates (in Å) of atoms in the optimized structure of the complex of linear D-ribose and Ag@CD.

C	-4.73393700	4.12824200	-0.17714700
C	-3.32335200	4.63441800	-0.44846300
C	-2.74579600	5.05521500	0.89929000
C	-3.85902500	3.31016800	2.04138700
C	-4.71005300	2.96515700	0.80597000
H	-2.71558100	3.80629000	-0.84967300
H	-5.31347200	4.95144400	0.27180300
H	-4.42984100	4.02219500	2.66003600
H	-4.27965600	2.09104100	0.31053300
H	-3.43426800	5.76181300	1.38741000
O	-5.34112800	3.74128000	-1.40166400
H	-5.15547400	4.47944300	-2.00929600
O	-3.43934900	5.69656400	-1.35770700
H	-2.53292500	5.93233100	-1.64026700
C	-3.53321000	2.08106300	2.87313000
H	-2.91536000	2.38780100	3.72418500
H	-4.46134100	1.63484700	3.23692600
O	-2.79400900	1.14517200	2.10306900
H	-3.30045700	0.30942300	1.94822900
O	-2.61020100	3.88784400	1.68230300
O	-6.03111800	2.70555400	1.27590700
O	-1.54125300	5.73678900	0.84320400
C	-0.38768200	5.06632200	0.35196300
C	0.18800800	5.90300800	-0.78061700
C	0.61330500	4.99305700	1.51591200
H	-0.63297700	4.06158900	-0.01989800
C	1.61132700	5.49669000	-1.14640400
H	0.21458100	6.95134300	-0.44171000
O	-0.65738100	5.78481500	-1.91217100
H	0.62976200	5.98852700	1.99014300
C	0.25797100	3.94790200	2.56223500
O	1.90511500	4.65004600	1.05661200
C	2.45989500	5.55699900	0.12685800
H	1.60376200	4.46643800	-1.53594700
O	2.04233000	6.39928900	-2.12944800
H	-0.16918200	6.19351800	-2.64813600
H	0.99800100	3.99474900	3.36608000
H	-0.73363400	4.14776000	2.97258400
O	0.34329800	2.63767200	2.02529700
H	2.44024900	6.58117800	0.52831500
H	2.88455900	6.04579200	-2.48064300
H	-0.39713100	2.46889100	1.41025800
O	3.80532900	5.25718200	-0.03251300
C	4.11255000	3.96569600	-0.53834100
C	4.97825700	4.11622800	-1.77249900
C	4.86644000	3.14695500	0.51851200
H	3.20926500	3.41694300	-0.80436100
C	5.35165600	2.73774200	-2.28670100
H	5.89051700	4.67691900	-1.50971500
O	4.26168500	4.81019500	-2.77658600
H	5.74952300	3.71659700	0.85113900
C	4.00673600	2.78940700	1.71787200
O	5.26351700	1.89289700	-0.02388300
C	6.06139400	1.93939900	-1.19227100
H	4.42038100	2.22887200	-2.56204900

O	6.17186600	2.91676800	-3.41524900
H	4.71858500	4.60594900	-3.61093300
H	4.62474000	2.24312100	2.44207000
H	3.62294600	3.69775500	2.18265100
O	2.95566000	1.94836700	1.26635100
H	7.03886200	2.39345300	-0.97620000
O	6.33777400	0.62343900	-1.55936100
H	6.11069100	2.08053900	-3.91486300
H	2.08177400	2.23622200	1.61067900
C	5.21149700	-0.18899800	-1.85886200
C	5.28494500	-0.65287400	-3.30193600
C	5.24492400	-1.42749600	-0.94457300
H	4.27299100	0.35541600	-1.70219900
C	4.14368400	-1.61813000	-3.58053800
H	6.24322200	-1.17706200	-3.45427800
O	5.19507500	0.44271100	-4.18910300
H	6.22427900	-1.91831000	-1.07241000
C	5.07164300	-1.09646100	0.52865700
O	4.20006200	-2.33243000	-1.28916400
C	4.26452600	-2.80534800	-2.62616700
H	3.19730700	-1.09031900	-3.38582100
O	4.25830900	-1.99743400	-4.92695500
H	4.95681900	0.05336600	-5.04925600
H	5.16362100	-2.01200900	1.12024500
H	5.83484900	-0.37798200	0.83402300
O	3.76723900	-0.57005800	0.77311900
H	5.22718000	-3.30885400	-2.79755200
H	3.38071600	-2.33853100	-5.18830400
H	3.75780100	0.40225900	0.93244300
O	3.31445900	-3.80161000	-2.81288100
C	1.93328700	-3.48795600	-2.67602400
C	1.22837700	-3.65171200	-4.01340200
C	1.36053600	-4.47593700	-1.64004000
H	1.79047600	-2.46059500	-2.33148400
C	-0.28334400	-3.64935000	-3.84631800
H	1.53471000	-4.61236500	-4.45713700
O	1.58797100	-2.58334900	-4.87609600
H	1.73572200	-5.47785700	-1.90591600
C	1.78912300	-4.14067500	-0.21796900
O	-0.05428100	-4.46667700	-1.62957800
C	-0.65668000	-4.76049200	-2.87290000
H	-0.58305100	-2.67860900	-3.42270500
O	-0.82510100	-3.83747400	-5.12880700
H	0.92183700	-2.59530800	-5.58736400
H	1.46963900	-4.93303500	0.46266300
H	2.87786600	-4.04092100	-0.16901600
O	1.14544200	-2.95042400	0.23056500
H	-0.30044900	-5.72865700	-3.25576400
O	-2.01942000	-4.93122200	-2.68543400
H	-1.77720800	-3.62316600	-5.06329100
H	1.40326700	-2.20510500	-0.35729100
C	-2.77380500	-3.84203500	-2.16520200
C	-3.90171400	-3.55590100	-3.14161200
C	-3.34635900	-4.27158100	-0.80897300
H	-2.15860900	-2.94512800	-2.01940600
C	-4.95714900	-2.63245900	-2.55288500
H	-4.38362500	-4.51464300	-3.39191900
O	-3.36588600	-2.96475300	-4.31458300

H	-3.79515100	-5.27181900	-0.92797900
C	-2.29553700	-4.31723300	0.28670100
O	-4.32424300	-3.34772000	-0.36509300
C	-5.43902700	-3.20886200	-1.22066600
H	-4.49316400	-1.65122600	-2.37404400
O	-5.98177500	-2.53992400	-3.50696000
H	-4.13452700	-2.65945400	-4.82711300
H	-2.78024900	-4.64102500	1.21652300
H	-1.51958900	-5.03485700	0.01688300
O	-1.76084600	-3.01466200	0.45905300
H	-5.91430500	-4.18559600	-1.39537600
H	-6.57187900	-1.81566700	-3.21600400
H	-0.78164100	-3.04143600	0.46691700
O	-6.40297800	-2.44679100	-0.57052100
C	-6.06299200	-1.10205300	-0.26862200
C	-7.10361400	-0.17660100	-0.86979500
C	-6.02726700	-0.88389500	1.24916500
H	-5.07628900	-0.83537900	-0.65801100
C	-6.70404700	1.24744800	-0.54009100
H	-8.09276900	-0.41688900	-0.44717100
O	-7.13617600	-0.32649700	-2.27746800
H	-7.00855500	-1.15183500	1.67435200
C	-4.94283000	-1.69653600	1.93705800
O	-5.71751400	0.47477100	1.53191000
C	-6.56720300	1.45587200	0.96515200
H	-5.72653800	1.40284500	-0.99923600
O	-7.65445200	2.12083300	-1.10689600
H	-7.55417700	0.48533400	-2.61338100
H	-5.00951300	-1.52923700	3.01937200
H	-5.09058900	-2.75591200	1.72272900
O	-3.68809500	-1.24265200	1.46494500
H	-7.55639600	1.43546700	1.44399800
H	-7.16869500	2.95090100	-1.26498800
H	-3.07431100	-1.96434000	1.21132300
Ag	0.75479800	-1.89293000	4.66454600
Ag	2.05069000	-1.66189800	2.23352600
Ag	3.25295900	-3.44209200	4.23185800
Ag	-0.60424700	-0.21192000	2.79361200
O	-0.10367500	-0.42208300	-2.71346400
O	-1.36738700	1.69139800	-0.09051500
O	1.94499600	-0.68484100	-1.09356800
O	-3.79072600	1.13648600	-1.63637800
O	2.22920600	1.82663900	-2.13193000
C	-0.28729600	-0.11613500	-1.34438300
C	-1.37462800	0.95525500	-1.29633600
C	1.03351500	0.31772000	-0.70766500
C	-2.69095500	0.25781000	-1.58417300
C	1.45997600	1.67666700	-1.21182700
H	-0.62143500	-1.01479700	-0.79400800
H	-1.15408700	1.66635300	-2.10738000
H	0.90665800	0.36847400	0.38589200
H	-2.90819800	-0.44909900	-0.76998600
H	-2.57731000	-0.31777100	-2.51476000
H	1.00652100	2.53967400	-0.68569100
H	0.82647500	-0.66416400	-2.85714100
H	-2.06450000	1.41281400	0.54820500
H	2.75186400	-0.64881900	-0.51794800
H	-3.65642300	1.81436800	-2.31562000

Table S9. The Cartesian coordinates (in Å) of atoms in the optimized structure of the complex of L-ribofuranose and Ag@CD.

C	2.42839300	-0.80959200	-2.62218800
H	3.26680100	-0.28258200	-3.09071400
H	2.46331400	-1.85358600	-2.97139600
C	1.12953100	-0.19253300	-3.10724600
H	0.99852100	-0.45360800	-4.16901900
O	1.19778800	1.22433800	-2.97671100
C	-0.02571100	1.69843700	-2.46112300
H	-0.75638400	1.85507300	-3.27146600
C	-0.13243900	-0.66116900	-2.34092400
H	0.07450400	-1.52154800	-1.68366400
C	-0.50004100	0.58319800	-1.53388500
H	0.13437800	0.61945800	-0.63432600
O	-1.86603200	0.63849500	-1.21939600
H	-1.98898000	1.31339700	-0.52527200
O	0.17854200	2.86800000	-1.73991100
H	0.38038100	3.61426600	-2.33395700
O	2.62391400	-0.71217900	-1.22663600
H	2.13521000	-1.41195000	-0.75368400
O	-1.20060400	-0.93814600	-3.21992300
H	-1.02168200	-1.74892700	-3.72859200
C	5.71407200	3.01878400	-0.74452200
C	6.01555400	1.55133700	-0.98464000
C	6.36882600	0.90220100	0.35232900
C	4.94051100	2.39230600	1.55366500
C	4.60497800	3.17677800	0.27853800
H	5.10733200	1.07595300	-1.38685400
H	6.62058200	3.51075900	-0.35631900
H	5.79594900	2.87825100	2.05109400
H	3.67195100	2.77672500	-0.13439700
H	7.26109900	1.38755300	0.77428900
O	5.32569000	3.62183800	-1.96522800
H	5.94956500	3.28706200	-2.63266100
O	7.07267400	1.50085700	-1.90636100
H	7.11528800	0.57764800	-2.22467000
C	3.77056100	2.30208100	2.52373100
H	4.06056800	1.63880400	3.34583500
H	3.53752700	3.29174500	2.92165700
O	2.64687100	1.72133300	1.88682200
H	2.03813700	2.42395500	1.58155100
O	5.26848700	1.04952400	1.23024800
O	4.50139500	4.55690500	0.60307300
O	6.68617000	-0.44427000	0.26890100
C	5.66365200	-1.30032300	-0.22427600
C	6.19674800	-2.04322000	-1.43585100
C	5.28691200	-2.30359600	0.87164700
H	4.75895200	-0.75187100	-0.50690400
C	5.17018500	-3.05941500	-1.89480100
H	7.13381500	-2.55448600	-1.16179000
O	6.43715900	-1.13021800	-2.49364200
H	6.20938600	-2.79306900	1.22865200
C	4.57546600	-1.67168900	2.06271500
O	4.38684800	-3.27101400	0.35977900
C	4.83172000	-4.01036000	-0.75032100
H	4.26799400	-2.49640300	-2.16057700
O	5.69489800	-3.72743900	-3.01401900

H	6.52897500	-1.67714400	-3.29316800
H	4.35247500	-2.46443000	2.78353800
H	5.23565000	-0.93953000	2.53408100
O	3.33835000	-1.09070300	1.69988900
H	5.71164000	-4.61987000	-0.49733200
H	4.93187300	-4.14029500	-3.46047800
H	3.46478800	-0.16786500	1.40695300
O	3.83126700	-4.92569100	-1.07469900
C	2.56025600	-4.38333600	-1.40284900
C	2.18486700	-4.87580600	-2.79192700
C	1.54074000	-4.82977200	-0.33967600
H	2.58448600	-3.29053900	-1.40191200
C	0.72603900	-4.58733100	-3.10175600
H	2.33734700	-5.96625300	-2.82902000
O	3.01754400	-4.24441000	-3.75016700
H	1.72265400	-5.89054300	-0.10830000
C	1.61778100	-3.99877600	0.92998600
O	0.20625300	-4.65216200	-0.79362700
C	-0.10408100	-5.27339100	-2.01966000
H	0.55633600	-3.49774800	-3.03413300
O	0.47056700	-5.06637400	-4.39279300
H	2.60763100	-4.42900500	-4.61357200
H	0.86099900	-4.36677400	1.63540800
H	2.60879900	-4.09162200	1.37439400
O	1.33150700	-2.64700700	0.58239100
H	0.14313100	-6.34490300	-1.98771700
O	-1.47268800	-5.22376700	-2.21674400
H	-0.41273100	-4.73390300	-4.64584000
H	1.83197800	-2.05355100	1.18754600
C	-2.09823700	-3.94665300	-2.16323200
C	-2.84754900	-3.74585600	-3.46588600
C	-3.04870700	-3.92755800	-0.96033500
H	-1.37380900	-3.13608500	-2.03401100
C	-3.82608600	-2.58845100	-3.40389200
H	-3.40219400	-4.66834500	-3.69460300
O	-1.89430900	-3.50185300	-4.50479600
H	-3.63867900	-4.85866400	-0.96583200
C	-2.32004800	-3.79697200	0.36833100
O	-3.91125700	-2.80706100	-1.03152000
C	-4.72761700	-2.76522000	-2.18559300
H	-3.25739900	-1.65918600	-3.28372600
O	-4.53612900	-2.59773800	-4.61802300
H	-2.42568900	-3.21620300	-5.27084900
H	-3.06714100	-3.70754200	1.16500900
H	-1.70695500	-4.68028600	0.55277900
O	-1.53854300	-2.60890500	0.37763100
H	-5.32034700	-3.68754100	-2.27320100
H	-4.96391100	-1.72120200	-4.68040300
H	-0.58543200	-2.80493900	0.27647200
O	-5.64780700	-1.74174600	-2.01969500
C	-5.10115200	-0.44773400	-1.80651200
C	-5.64554900	0.48358000	-2.87281300
C	-5.53402000	0.05140900	-0.41969800
H	-4.00498800	-0.44197400	-1.85965100
C	-5.17750800	1.89933000	-2.59439200
H	-6.74719000	0.45220900	-2.84402500
O	-5.18825300	0.07763000	-4.14907900
H	-6.63220700	-0.03262600	-0.35391700

C	-4.92177200	-0.72189700	0.74106700
O	-5.13255900	1.39875100	-0.24642900
C	-5.64979300	2.30445200	-1.19785300
H	-4.07710000	1.89814400	-2.62412600
O	-5.71750700	2.71805600	-3.59921100
H	-5.32580400	0.84702400	-4.72878500
H	-5.30226700	-0.31318300	1.68068000
H	-5.18952700	-1.77871900	0.67512400
O	-3.51103500	-0.55451700	0.78419300
H	-6.74952400	2.30913900	-1.17172700
O	-5.27070900	3.58651100	-0.82127400
H	-5.22137100	3.55887100	-3.56117900
H	-3.08933300	-1.01656900	0.03082800
C	-3.87682900	3.85141400	-0.76583700
C	-3.57246700	5.01073300	-1.69504300
C	-3.50836800	4.21223300	0.68129400
H	-3.28286000	2.98159400	-1.06730700
C	-2.13170400	5.44212100	-1.51298700
H	-4.24174800	5.85032300	-1.44641500
O	-3.77530700	4.61453400	-3.03932200
H	-4.23174600	4.96102400	1.04330600
C	-3.52297000	3.00944700	1.60696500
O	-2.18951200	4.72851300	0.76369200
C	-1.92238400	5.84662400	-0.05669700
H	-1.49077000	4.57619000	-1.72858000
O	-1.89188400	6.49527500	-2.41216100
H	-3.32901800	5.29191600	-3.57703300
H	-3.24749100	3.34279500	2.61489300
H	-4.52573600	2.57977100	1.62666000
O	-2.56707600	2.06111600	1.14892100
H	-2.58670700	6.68543400	0.19912500
H	-0.92283100	6.54936100	-2.50642300
H	-2.96063100	1.16159400	1.16697600
O	-0.63934300	6.29400600	0.22206600
C	0.40767700	5.33880100	0.07530500
C	1.37370600	5.83998400	-0.98293300
C	1.09383400	5.20373400	1.44115700
H	0.02546000	4.35436400	-0.20512600
C	2.69147400	5.08914200	-0.96364600
H	1.57486700	6.90603900	-0.79718400
O	0.79465600	5.69359800	-2.28290000
H	1.24489500	6.21112600	1.86153100
C	0.27340300	4.35307500	2.40405900
O	2.34328000	4.53879700	1.32517900
C	3.26264400	5.15892000	0.44977700
H	2.49149600	4.03787900	-1.22139200
O	3.50900900	5.71224000	-1.91989200
H	1.52836100	5.88252000	-2.89743200
H	0.81993900	4.28496700	3.35183300
H	-0.69344200	4.82824700	2.58127100
O	0.13489500	3.04422400	1.88731500
H	3.41564200	6.20948300	0.73776500
H	4.28452900	5.13051900	-2.05325800
H	-0.77175500	2.87947800	1.56336300
Ag	-1.68955000	-1.21904300	2.40956100
Ag	-0.62604800	-1.16312700	4.95124000
Ag	-3.02694900	-2.83410500	4.57550900
Ag	0.76628100	0.35442000	2.94067600

Table S10. The Cartesian coordinates (in Å) of atoms in the optimized structure of the complex of D-ribofuranose and Ag@CD.

C	-0.94563800	2.56658900	-2.29605700
H	-0.33194600	3.06329000	-3.05605500
H	-1.85689600	3.17138300	-2.16340100
C	-1.35808500	1.20042300	-2.81331800
H	-2.08283400	1.32953100	-3.62798200
O	-0.20512100	0.51215100	-3.30753900
C	-0.18027100	-0.79654800	-2.79683700
H	-0.74770900	-1.48258500	-3.44930800
C	-1.99206200	0.29330700	-1.74539600
H	-2.31411000	0.85143300	-0.85189600
C	-0.86882300	-0.69714900	-1.43680600
H	-0.16089900	-0.26842300	-0.71345500
O	-1.41159400	-1.91756000	-0.99964400
H	-0.77999000	-2.35316500	-0.37733300
O	1.13270300	-1.24391900	-2.65877000
H	1.50497800	-1.44353600	-3.53670500
O	-0.16883100	2.51444500	-1.11766100
H	-0.73861600	2.39814000	-0.33009200
O	-3.09267000	-0.38989300	-2.31654000
H	-3.05677800	-1.30986900	-2.00241400
C	-6.19692500	2.02755100	-0.55618200
C	-5.21467100	3.17994800	-0.68887900
C	-4.96801800	3.75450100	0.70621600
C	-5.26638900	1.61938700	1.73762800
C	-5.65484200	0.97841100	0.39961900
H	-4.27191500	2.77179800	-1.08953300
H	-7.14550400	2.41607500	-0.15067200
H	-6.18660100	1.92613700	2.26178900
H	-4.75436100	0.52746800	-0.03352500
H	-5.91955800	4.12073500	1.11995000
O	-6.41956100	1.44211100	-1.82439400
H	-6.50844100	2.18478000	-2.44644200
O	-5.78460900	4.11085800	-1.56857500
H	-5.08389700	4.75521700	-1.79295500
C	-4.45302000	0.69486500	2.63102000
H	-4.18498500	1.24509000	3.54002700
H	-5.04387200	-0.18138000	2.90018600
O	-3.25653600	0.32266900	1.96426700
H	-3.25054700	-0.64060000	1.77139000
O	-4.42812900	2.74107700	1.52111700
O	-6.67641800	0.01624700	0.62936500
O	-4.11447900	4.84729600	0.74783700
C	-2.79548800	4.67952800	0.24443600
C	-2.57583100	5.76381600	-0.79387300
C	-1.79063300	4.77731500	1.40516600
H	-2.66598200	3.69982600	-0.22517300
C	-1.11550200	5.89274200	-1.17774500
H	-2.91299300	6.72320000	-0.37004600
O	-3.34503300	5.45098500	-1.94525300
H	-2.08771300	5.61429800	2.05645100
C	-1.71183200	3.49177100	2.21868100
O	-0.46950700	4.98932100	0.92292000
C	-0.30539100	6.12532000	0.09099200
H	-0.76997800	4.95139600	-1.62214800
O	-1.02592800	6.95089400	-2.09550200

H	-3.02090100	6.04737600	-2.64294200
H	-0.87570900	3.58247200	2.92289000
H	-2.64032700	3.35282000	2.77299800
O	-1.47064500	2.39640500	1.34714600
H	-0.64946300	7.03277400	0.60865900
H	-0.15856600	6.84930100	-2.53334200
H	-2.10624100	1.67438000	1.54002100
O	1.04844600	6.31684800	-0.13843600
C	1.75329200	5.19681400	-0.66210400
C	2.36543100	5.55343300	-2.00272200
C	2.86147700	4.84546200	0.33807500
H	1.09952600	4.32612900	-0.79215700
C	3.24088500	4.40408400	-2.47146900
H	2.98283000	6.45961000	-1.89166700
O	1.34521000	5.78272100	-2.95988800
H	3.44849400	5.75747700	0.53873800
C	2.32309300	4.29702000	1.65221800
O	3.69976500	3.83085300	-0.18687600
C	4.31681300	4.14103000	-1.41736300
H	2.60231800	3.51255500	-2.56916900
O	3.78660600	4.77435700	-3.71068400
H	1.79004300	5.73551100	-3.82464800
H	3.16732800	4.08572500	2.31488800
H	1.67506400	5.04054900	2.12443800
O	1.64799800	3.06898400	1.46051100
H	4.95551200	5.03098800	-1.31755800
O	5.18179000	3.10422200	-1.73753200
H	4.13398400	3.95285300	-4.11046000
H	0.75570700	3.24836700	1.11394100
C	4.60785900	1.81303600	-1.89124700
C	4.96432000	1.30992200	-3.27852700
C	5.19024700	0.89160900	-0.80978100
H	3.51776800	1.83019000	-1.77810600
C	4.58640500	-0.15218400	-3.43026800
H	6.05332600	1.40960400	-3.41658100
O	4.28473300	2.08302800	-4.25010400
H	6.28419900	1.02047000	-0.79160700
C	4.62361600	1.15969200	0.57678300
O	4.87279200	-0.46764400	-1.08695200
C	5.31700700	-0.93891300	-2.34377900
H	3.50026100	-0.24626600	-3.27386500
O	4.95820400	-0.53906900	-4.72765400
H	4.35310100	1.57966800	-5.07988900
H	5.04913300	0.42745500	1.27330300
H	4.89212900	2.16580200	0.90266200
O	3.21405500	0.97986000	0.56232500
H	6.40210400	-0.79373600	-2.44563300
H	4.51348100	-1.39110000	-4.89711300
H	2.74617100	1.84364000	0.62406200
O	5.12798900	-2.31266200	-2.39435500
C	3.79962400	-2.78039300	-2.20351400
C	3.39101400	-3.58032400	-3.42444000
C	3.75751900	-3.65024900	-0.93992400
H	3.09306500	-1.95843800	-2.06626300
C	2.10467600	-4.34839000	-3.19871600
H	4.19863300	-4.28419000	-3.67538600
O	3.17704900	-2.68510000	-4.51760200
H	4.56891100	-4.39478400	-0.98714000

C	3.88913200	-2.82242800	0.33232700
O	2.49956000	-4.29710900	-0.83717100
C	2.17965200	-5.15945800	-1.90850800
H	1.30117100	-3.60958900	-3.09159300
O	1.90574900	-5.14696600	-4.33741800
H	2.79220500	-3.23797400	-5.22176900
H	3.76306600	-3.47358000	1.20190200
H	4.86993100	-2.34487100	0.38320300
O	2.84751800	-1.85756000	0.38774900
H	2.93208800	-5.95576400	-2.00350200
O	0.99053900	-5.80662200	-1.59707000
H	0.95750200	-5.38369500	-4.33581000
H	3.12023100	-1.06565700	-0.11762800
C	-0.16563800	-4.99065600	-1.44458300
C	-1.20207800	-5.44594300	-2.45520500
C	-0.68071700	-5.15851200	-0.00532800
H	0.05562600	-3.93078200	-1.60733500
C	-2.55224200	-4.81613800	-2.17294700
H	-1.30154900	-6.54108200	-2.38134400
O	-0.78815400	-5.09018000	-3.76351600
H	-0.70635000	-6.23425500	0.23121000
C	0.19691200	-4.44310800	1.01319100
O	-1.98955700	-4.61009000	0.13720700
C	-2.94687000	-5.18070800	-0.74608800
H	-2.45397000	-3.72357900	-2.24695300
O	-3.44150700	-5.32712600	-3.13167200
H	-1.58154000	-5.19942500	-4.31744200
H	-0.26456300	-4.54355500	2.00187800
H	1.18487800	-4.90639000	1.02532800
O	0.28776200	-3.05988000	0.70915400
H	-2.95638100	-6.27416600	-0.62841700
H	-4.21619300	-4.73212900	-3.12588800
H	1.20128700	-2.80481800	0.45886700
O	-4.21886300	-4.77042100	-0.38082500
C	-4.46094200	-3.37173800	-0.32015800
C	-5.54424300	-2.98775100	-1.31081500
C	-4.92975900	-3.05563400	1.10274300
H	-3.54995100	-2.79532800	-0.52627200
C	-5.96180300	-1.54124400	-1.10589000
H	-6.41438600	-3.64573300	-1.15788100
O	-5.05216200	-3.13192800	-2.63407700
H	-5.78700600	-3.70781200	1.34012200
C	-3.83342900	-3.27200200	2.13464500
O	-5.30388800	-1.69506600	1.19994900
C	-6.36516100	-1.30687700	0.34793000
H	-5.09892600	-0.90744700	-1.34598900
O	-7.01710500	-1.28706500	-1.99906300
H	-5.66237400	-2.62032600	-3.19517400
H	-4.22341700	-3.01934000	3.12452500
H	-3.52045500	-4.31966700	2.12740400
O	-2.73575900	-2.40927100	1.89537400
H	-7.27438000	-1.87935100	0.58192800
H	-7.07191500	-0.31433200	-2.07714400
H	-2.16443600	-2.81225800	1.21702100
Ag	1.33680500	-1.07268200	4.86549800
Ag	2.11066900	-0.31308000	2.31290800
Ag	4.19154100	-1.25353600	4.29885600
Ag	-0.75063700	-0.41333000	3.00470900
